# *PHIP* - a novel candidate breast cancer susceptibility locus on 6q14.1

**DOI:** 10.18632/oncotarget.21800

**Published:** 2017-10-12

**Authors:** Xiang Jiao, Christos Aravidis, Rajeshwari Marikkannu, Johanna Rantala, Simone Picelli, Tatjana Adamovic, Tao Liu, Paula Maguire, Barbara Kremeyer, Liping Luo, Susanna von Holst, Vinaykumar Kontham, Jessada Thutkawkorapin, Sara Margolin, Quan Du, Johanna Lundin, Kyriaki Michailidou, Manjeet K. Bolla, Qin Wang, Joe Dennis, Michael Lush, Christine B. Ambrosone, Irene L. Andrulis, Hoda Anton-Culver, Natalia N. Antonenkova, Volker Arndt, Matthias W. Beckmann, Carl Blomqvist, William Blot, Bram Boeckx, Stig E. Bojesen, Bernardo Bonanni, Judith S. Brand, Hiltrud Brauch, Hermann Brenner, Annegien Broeks, Thomas Brüning, Barbara Burwinkel, Qiuyin Cai, Jenny Chang-Claude, NBCS Collaborators, Fergus J. Couch, Angela Cox, Simon S. Cross, Sandra L. Deming-Halverson, Peter Devilee, Isabel dos-Santos-Silva, Thilo Dörk, Mikael Eriksson, Peter A. Fasching, Jonine Figueroa, Dieter Flesch-Janys, Henrik Flyger, Marike Gabrielson, Montserrat García-Closas, Graham G. Giles, Anna González-Neira, Pascal Guénel, Qi Guo, Melanie Gündert, Christopher A. Haiman, Emily Hallberg, Ute Hamann, Patricia Harrington, Maartje J. Hooning, John L. Hopper, Guanmengqian Huang, Anna Jakubowska, Michael E. Jones, Michael J. Kerin, Veli-Matti Kosma, Vessela N. Kristensen, Diether Lambrechts, Loic Le Marchand, Jan Lubinski, Arto Mannermaa, John W.M. Martens, Alfons Meindl, Roger L. Milne, Anna Marie Mulligan, Susan L. Neuhausen, Heli Nevanlinna, Julian Peto, Katri Pylkäs, Paolo Radice, Valerie Rhenius, Elinor J. Sawyer, Marjanka K. Schmidt, Rita K. Schmutzler, Caroline Seynaeve, Mitul Shah, Jacques Simard, Melissa C. Southey, Anthony J. Swerdlow, Thérèse Truong, Camilla Wendt, Robert Winqvist, Wei Zheng, kConFab/AOCS Investigators, Javier Benitez, Alison M. Dunning, Paul D.P. Pharoah, Douglas F. Easton, Kamila Czene, Per Hall, Annika Lindblom

**Affiliations:** ^1^ Department of Molecular Medicine and Surgery, Karolinska Institutet, Stockholm, Sweden; ^2^ Department of Immunology, Genetics and Pathology, Uppsala University, Uppsala, Sweden; ^3^ Department of Oncology - Pathology, Karolinska Institutet, Stockholm, Sweden; ^4^ Centre for Cancer Genetic Epidemiology, Department of Public Health and Primary Care, University of Cambridge, Cambridge, UK; ^5^ Department of Electron Microscopy/Molecular Pathology, The Cyprus Institute of Neurology and Genetics, Nicosia, Cyprus; ^6^ Roswell Park Cancer Institute, Buffalo, NY, USA; ^7^ Fred A. Litwin Center for Cancer Genetics, Lunenfeld-Tanenbaum Research Institute of Mount Sinai Hospital, Toronto, ON, Canada; ^8^ Department of Molecular Genetics, University of Toronto, Toronto, ON, Canada; ^9^ Department of Epidemiology, University of California Irvine, Irvine, CA, USA; ^10^ N.N. Alexandrov Research Institute of Oncology and Medical Radiology, Minsk, Belarus; ^11^ Division of Clinical Epidemiology and Aging Research, German Cancer Research Center (DKFZ), Heidelberg, Germany; ^12^ Department of Gynaecology and Obstetrics, University Hospital Erlangen, Friedrich-Alexander University Erlangen-Nuremberg, Comprehensive Cancer Center Erlangen-EMN, Erlangen, Germany; ^13^ Department of Oncology, Helsinki University Hospital, University of Helsinki, Helsinki, Finland; ^14^ Division of Epidemiology, Department of Medicine, Vanderbilt Epidemiology Center, Vanderbilt-Ingram Cancer Center, Vanderbilt University School of Medicine, Nashville, TN, USA; ^15^ International Epidemiology Institute, Rockville, MD, USA; ^16^ VIB Center for Cancer Biology, VIB, Leuven, Belgium; ^17^ Laboratory for Translational Genetics, Department of Human Genetics, University of Leuven, Leuven, Belgium; ^18^ Copenhagen General Population Study, Herlev and Gentofte Hospital, Copenhagen University Hospital, Herlev, Denmark; ^19^ Department of Clinical Biochemistry, Herlev and Gentofte Hospital, Copenhagen University Hospital, Herlev, Denmark; ^20^ Faculty of Health and Medical Sciences, University of Copenhagen, Copenhagen, Denmark; ^21^ Division of Cancer Prevention and Genetics, Istituto Europeo di Oncologia, Milan, Italy; ^22^ Department of Medical Epidemiology and Biostatistics, Karolinska Institutet, Stockholm, Sweden; ^23^ Dr. Margarete Fischer-Bosch-Institute of Clinical Pharmacology, Stuttgart, Germany; ^24^ University of Tübingen, Tübingen, Germany; ^25^ German Cancer Consortium (DKTK), German Cancer Research Center (DKFZ), Heidelberg, Germany; ^26^ Division of Preventive Oncology, German Cancer Research Center (DKFZ) and National Center for Tumor Diseases (NCT), Heidelberg, Germany; ^27^ Division of Molecular Pathology, The Netherlands Cancer Institute - Antoni van Leeuwenhoek Hospital, Amsterdam, The Netherlands; ^28^ Institute for Prevention and Occupational Medicine of the German Social Accident Insurance, Institute of the Ruhr University Bochum, Bochum, Germany; ^29^ Department of Obstetrics and Gynecology, University of Heidelberg, Heidelberg, Germany; ^30^ Molecular Epidemiology Group, C080, German Cancer Research Center (DKFZ), Heidelberg, Germany; ^31^ Division of Cancer Epidemiology, German Cancer Research Center (DKFZ), Heidelberg, Germany; ^32^ Research Group Genetic Cancer Epidemiology, University Cancer Center Hamburg (UCCH), University Medical Center Hamburg-Eppendorf, Hamburg, Germany; ^33^ Department of Oncology, Haukeland University Hospital, Bergen, Norway; ^34^ Section of Oncology, Institute of Medicine, University of Bergen, Bergen, Norway; ^35^ Department of Pathology, Akershus University Hospital, Lørenskog, Norway; ^36^ Department of Breast-Endocrine Surgery, Akershus University Hospital, Lørenskog, Norway; ^37^ Department of Cancer Genetics, Institute for Cancer Research, Oslo University Hospital Radiumhospitalet, Oslo, Norway; ^38^ Department of Breast and Endocrine Surgery, Oslo University Hospital, Ullevål, Oslo, Norway; ^39^ Department of Research, Vestre Viken Hospital, Drammen, Norway; ^40^ Department of Tumor Biology, Institute for Cancer Research, Oslo University Hospital Radiumhospitalet, Oslo, Norway; ^41^ Institute of Clinical Medicine, Faculty of Medicine, University of Oslo, Oslo, Norway; ^42^ Department of Clinical Molecular Biology, Oslo University Hospital, University of Oslo, Oslo, Norway; ^43^ National Advisory Unit on Late Effects after Cancer Treatment, Oslo University Hospital Radiumhospitalet, Oslo, Norway; ^44^ Department of Oncology, Oslo University Hospital Radiumhospitalet, Oslo, Norway; ^45^ Department of Radiology and Nuclear Medicine, Oslo University Hospital Radiumhospitalet, Oslo, Norway; ^46^ Oslo University Hospital, Oslo, Norway; ^47^ Department of Oncology, Oslo University Hospital Ullevål, Oslo, Norway; ^48^ Department of Laboratory Medicine and Pathology, Mayo Clinic, Rochester, MN, USA; ^49^ Sheffield Institute for Nucleic Acids (SInFoNiA), Department of Oncology and Metabolism, University of Sheffield, Sheffield, UK; ^50^ Academic Unit of Pathology, Department of Neuroscience, University of Sheffield, Sheffield, UK; ^51^ Department of Pathology, Leiden University Medical Center, Leiden, The Netherlands; ^52^ Department of Human Genetics, Leiden University Medical Center, Leiden, The Netherlands; ^53^ Department of Non-Communicable Disease Epidemiology, London School of Hygiene and Tropical Medicine, London, UK; ^54^ Gynaecology Research Unit, Hannover Medical School, Hannover, Germany; ^55^ David Geffen School of Medicine, Department of Medicine Division of Hematology and Oncology, University of California at Los Angeles, Los Angeles, CA, USA; ^56^ Usher Institute of Population Health Sciences and Informatics, The University of Edinburgh Medical School, Edinburgh, UK; ^57^ Division of Cancer Epidemiology and Genetics, National Cancer Institute, Rockville, MD, USA; ^58^ Institute for Medical Biometrics and Epidemiology, University Medical Center Hamburg-Eppendorf, Hamburg, Germany; ^59^ Department of Cancer Epidemiology, Clinical Cancer Registry, University Medical Center Hamburg-Eppendorf, Hamburg, Germany; ^60^ Department of Breast Surgery, Herlev and Gentofte Hospital, Copenhagen University Hospital, Herlev, Denmark; ^61^ Cancer Epidemiology & Intelligence Division, Cancer Council Victoria, Melbourne, Victoria, Australia; ^62^ Centre for Epidemiology and Biostatistics, Melbourne School of Population and Global health, The University of Melbourne, Melbourne, Victoria, Australia; ^63^ Human Cancer Genetics Program, Spanish National Cancer Research Centre, Madrid, Spain; ^64^ Cancer & Environment Group, Center for Research in Epidemiology and Population Health (CESP), INSERM, University Paris-Sud, University Paris-Saclay, Villejuif, France; ^65^ Cardiovascular Epidemiology Unit, Department of Public Health and Primary Care, University of Cambridge, Cambridge, UK; ^66^ Department of Preventive Medicine, Keck School of Medicine, University of Southern California, Los Angeles, CA, USA; ^67^ Department of Health Sciences Research, Mayo Clinic, Rochester, MN, USA; ^68^ Molecular Genetics of Breast Cancer, German Cancer Research Center (DKFZ), Heidelberg, Germany; ^69^ Centre for Cancer Genetic Epidemiology, Department of Oncology, University of Cambridge, Cambridge, UK; ^70^ Department of Medical Oncology, Family Cancer Clinic, Erasmus MC Cancer Institute, Rotterdam, The Netherlands; ^71^ Department of Genetics and Pathology, Pomeranian Medical University, Szczecin, Poland; ^72^ Division of Genetics and Epidemiology, The Institute of Cancer Research, London, UK; ^73^ School of Medicine, National University of Ireland, Galway, Ireland; ^74^ Translational Cancer Research Area, University of Eastern Finland, Kuopio, Finland; ^75^ Institute of Clinical Medicine, Pathology and Forensic Medicine, University of Eastern Finland, Kuopio, Finland; ^76^ Imaging Center, Department of Clinical Pathology, Kuopio University Hospital, Kuopio, Finland; ^77^ Epidemiology Program, University of Hawaii Cancer Center, Honolulu, HI, USA; ^78^ Division of Gynaecology and Obstetrics, Technische Universität München, Munich, Germany; ^79^ Department of Laboratory Medicine and Pathobiology, University of Toronto, Toronto, ON, Canada; ^80^ Laboratory Medicine Program, University Health Network, Toronto, ON, Canada; ^81^ Department of Population Sciences, Beckman Research Institute of City of Hope, Duarte, CA, USA; ^82^ Department of Obstetrics and Gynecology, Helsinki University Hospital, University of Helsinki, Helsinki, Finland; ^83^ Laboratory of Cancer Genetics and Tumor Biology, Cancer and Translational Medicine Research Unit, Biocenter Oulu, University of Oulu, Oulu, Finland; ^84^ Laboratory of Cancer Genetics and Tumor Biology, Northern Finland Laboratory Centre Oulu, Oulu, Finland; ^85^ Department of Research, Fondazione IRCCS (Istituto Di Ricovero e Cura a Carattere Scientifico) Istituto Nazionale dei Tumori (INT), Milan, Italy; ^86^ Research Oncology, Guy’s Hospital, King’s College London, London, UK; ^87^ Division of Psychosocial Research and Epidemiology, The Netherlands Cancer Institute - Antoni van Leeuwenhoek hospital, Amsterdam, The Netherlands; ^88^ Center for Hereditary Breast and Ovarian Cancer, University Hospital of Cologne, Cologne, Germany; ^89^ Center for Integrated Oncology (CIO), University Hospital of Cologne, Cologne, Germany; ^90^ Center for Molecular Medicine Cologne (CMMC), University of Cologne, Cologne, Germany; ^91^ Genomics Center, Centre Hospitalier Universitaire de Québec Research Center, Laval University, Québec City, QC, Canada; ^92^ Department of Pathology, The University of Melbourne, Melbourne, Victoria, Australia; ^93^ Division of Breast Cancer Research, The Institute of Cancer Research, London, UK; ^94^ Peter MacCallum Cancer Center, Melbourne, Victoria, Australia; ^95^ The Sir Peter MacCallum Department of Oncology University of Melbourne, Parkville, Australia; ^96^ Centro de Investigación en Red de Enfermedades Raras (CIBERER), Valencia, Spain

**Keywords:** familial breast cancer, linkage analysis, risk haplotype, sequencing

## Abstract

Most non-*BRCA1/2* breast cancer families have no identified genetic cause. We used linkage and haplotype analyses in familial and sporadic breast cancer cases to identify a susceptibility locus on chromosome 6q. Two independent genome-wide linkage analysis studies suggested a 3 Mb locus on chromosome 6q and two unrelated Swedish families with a LOD >2 together seemed to share a haplotype in 6q14.1. We hypothesized that this region harbored a rare high-risk founder allele contributing to breast cancer in these two families. Sequencing of DNA and RNA from the two families did not detect any pathogenic mutations. Finally, 29 SNPs in the region were analyzed in 44,214 cases and 43,532 controls from BCAC, and the original haplotypes in the two families were suggested as low-risk alleles for European and Swedish women specifically. There was also some support for one additional independent moderate-risk allele in Swedish familial samples. The results were consistent with our previous findings in familial breast cancer and supported a breast cancer susceptibility locus at 6q14.1 around the *PHIP* gene.

## INTRODUCTION

Only 5% of all breast cancer cases are attributed to the segregation of germline mutations in high penetrance genes within families [1-3]. The two breast cancer genes *BRCA1* and *BRCA2*, account for 10-15% of the familial risk of breast cancer, while mutations in other high-risk genes *PTEN*, *STK11*, *CDH1*, and *TP53* or in the moderate-risk genes *ATM*, *CHEK2*, *PALB2*, explain around 5% of familial cases [4]. Thus, the great majority of families remain unexplained. Linkage analysis followed by positional cloning has been successfully applied to identify the high penetrance breast cancer susceptibility genes *BRCA1* and *BRCA2* [5, 6]. Subsequent linkage studies have been performed in non-*BRCA1/2* families, without identifying any novel putative breast cancer genes [7-14]. Most other high- or moderate risk genes/alleles have been identified through a candidate gene approach [15-20], while others have been identified through their contribution to other cancer syndromes [21, 22]. Genome-wide association studies, mostly within the Breast Cancer Association Consortium (BCAC), have identified low-penetrance alleles that explain part of the remaining familial breast cancer risk [23]. In total, these and other studies so far have identified 94 genetic low risk loci, each with a typical relative risk (RR) of less than 1.2, estimated to contribute in total 14% of the remaining familial risk of breast cancer [23]. Over the past a few years, whole-exome sequencing has been utilized to search for novel breast cancer susceptibility genes, assuming that the missing breast cancer heritability can partly be attributed to rare risk alleles segregating in families in an autosomal-dominant pattern (reviewed in [24]). Despite a handful of genes being reported using this technology, the vast majority of exome-sequenced cancer families remain unsolved [24]. There is evidence that there are more high- and low risk alleles to be identified, and that up to 28% of the familial risk could be attributed to still unidentified risk SNPs [23]. The remaining risk alleles could be rare and associated with smaller risks and would thus be difficult to find. Nevertheless, it is possible that a traditional strategy such as linkage analysis still might give a lead to new candidate susceptibility loci. We have used linkage and association studies in familial and sporadic breast cancer to define a breast cancer susceptibility locus on chromosome 6q.

## RESULTS

### Linkage analysis revealed a 2.8 Mb linked region in breast cancer families

**Figure 1:**
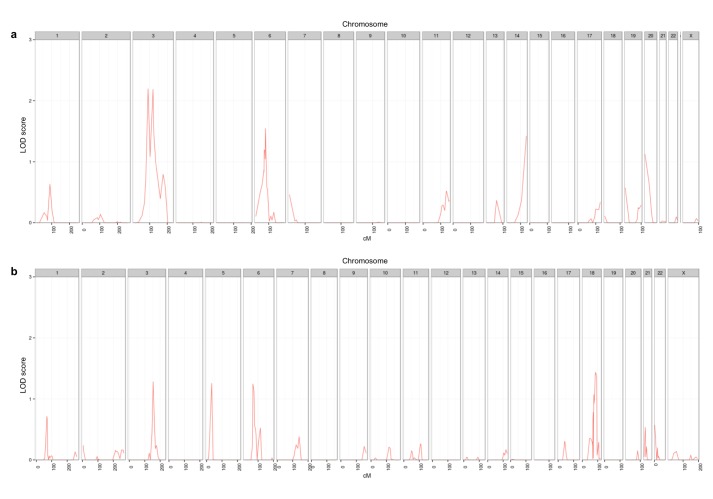
Linkage analysis revealed genomic regions with HLOD score above one in: a) families with moderate risk of breast cancer and b) High risk breast cancer families with other types of cancer

In an attempt to identify new breast cancer susceptibility loci, a genome-wide linkage analysis was conducted in 96 non-BRCA1/2 families with breast or breast-ovarian cancer. For most families, there were few individuals available for study and no overall significant positive logarithm of odds (LOD), heterogeneity LOD (HLOD) or nonparametric linkage LOD (NPL LOD) scores were obtained for any chromosome [25]. We assumed that the families were heterogeneous and likely to segregate different risk alleles. Thus, we analyzed separately families with at least three affected women (high-risk), families with only two cases (moderate-risk) and families with breast cancer and other types of cancer (putative breast cancer syndromes). There were weakly positive LOD scores for the subset of 22 moderate-risk families, which showed HLODs above 1 for regions on chromosomes 3, 6 and 14 (Figure 1a). Fine-mapping of those three regions confirmed and defined the regions to 3q25, 6q14 and 14q32. By reanalyzing a previously published linkage analysis in 14 large high-risk breast cancer families [26] but coding all cancers to define affected status, three additional loci showed HLOD scores above one, on chromosomes 3, 5 and 6 (Figure 1b). Both regions on chromosome 3q and 6q overlapped with those obtained in the previous analysis in moderate-risk families.

**Figure 2:**
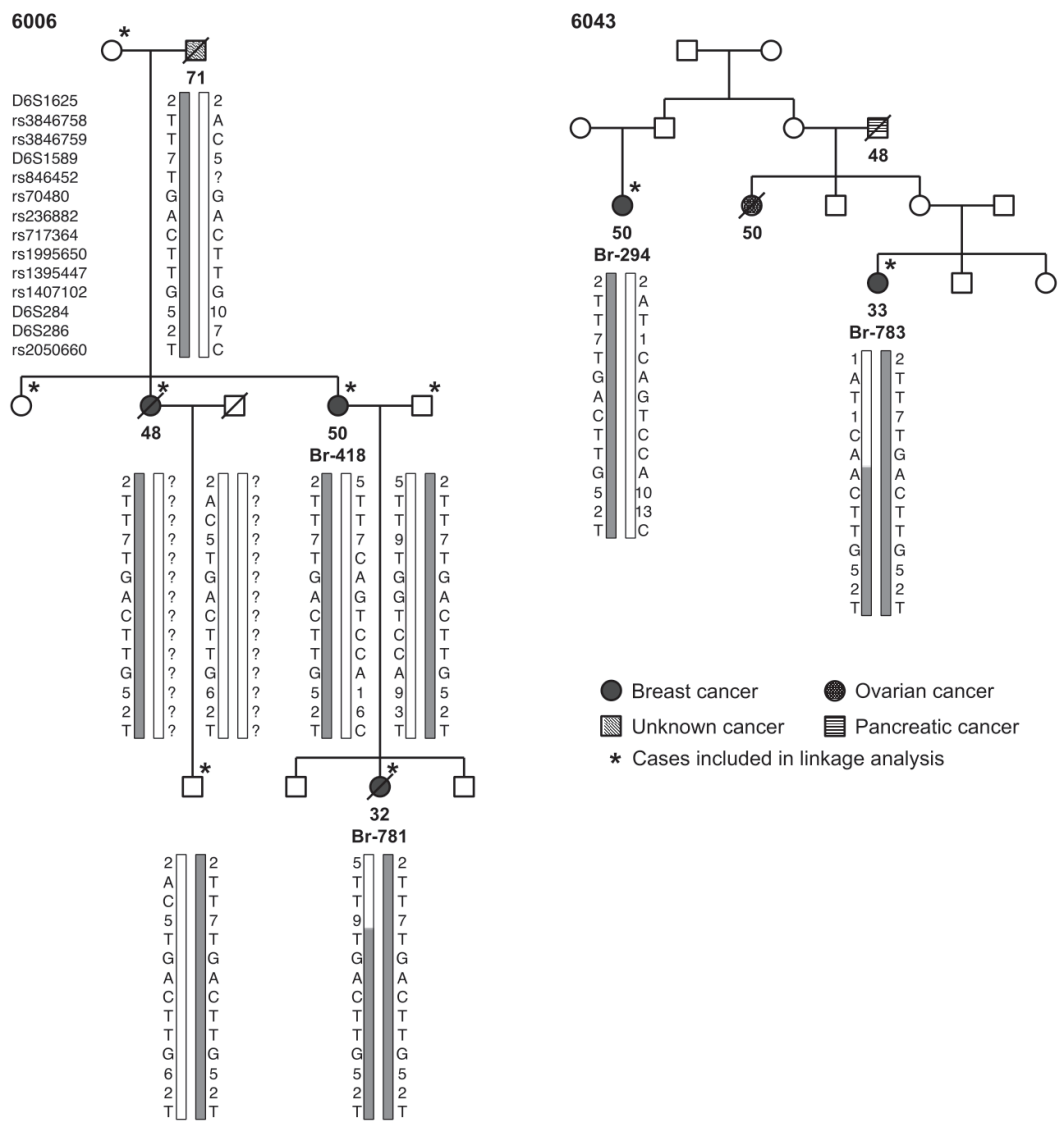
Pedigrees of the families 6006 and 6043 showing the haplotypes of selected family members (the families’ risk haplotype is indicated in grey shade)

Fine-mapping of the 6q region was conducted using a total of 43 families. Only two unrelated families, 6006 and 6043, shared a unique 2.8 Mb haplotype between the markers D6S1625 and rs2050660 on chromosome 6q14.1 (Figure 2). Max LOD scores of 1.48 for family 6006 and 0.78 for family 6043 were observed, which were among the highest for the families included in the linkage analysis. To assess whether the two families are related, we searched the linkage data for other shared and possibly linked regions and identified two more, one on chromosome 7 and one on chromosome 10. Fine-mapping of both regions (data not shown) demonstrated that the only linked region shared was on chromosome 6q. Thus, the families were considered not closely related. The finding of a shared haplotype in affected cases in both families together with the overall weak support for a region on 6q was consistent with a susceptibility locus within the region defined by the linkage studies, and in particular by the 2.8 Mb shared region flanked by D6S1625 and rs2050660. The locus on chromosome 6 was chosen for further studies.

### Mutation screening of the 6q candidate region found no clear pathogenic mutation

A mutation screen of one affected member from each of 31 families (including 6006 and 6043) with positive LOD scores for the region revealed no clear pathogenic mutation in any of the six genes within this region. RT-PCR of mRNA from two cases from each of the two families 6006 and 6043 also did not detect any pathogenic mutation. Array comparative genomic hybridization (array-CGH) was performed in the two families, but no copy number alteration was observed in the candidate region. Eleven common variants were identified in the two families (Table 1).

**Table 1:** Sequence variants shared by families 6006 and 6043 from Sanger sequencing.

Genomic variants^a^	dbSNP ID	Gene	Variant type	MAF^b^
chr 6: 79595097 A>G	rs6908105	*IRAK1BP1*	Synonymous	0.27
chr 6: 79656562 A>T	rs2275291	*PHIP*	Synonymous	0.24
chr 6: 79656570 C>T	rs2275290	*PHIP*	Synonymous	0.28
chr 6: 79657391 C>T	rs1984195	*PHIP*	Synonymous	0.51
chr 6: 79664440 A>G	rs9443632	*PHIP*	Intronic	0.52
chr 6: 79664748 A>G	rs10455356	*PHIP*	Intronic	0.52
chr 6: 79675701 T>C	rs9350797	*PHIP*	Missense p.L1093P	0.22
chr 6: 79679577 T>C	rs7742431	*PHIP*	Synonymous	0.55
chr 6: 79695029 G>A	rs1890229	*PHIP*	Intronic	0.52
chr 6: 79707923 G>A	rs11752126	*PHIP*	Intronic	0.39
chr 6: 79752792 T>C	rs9343863	*PHIP*	Intronic	0.51

^a^ Genomic coordinates were based on GRCh37.

^b^ Minor allele frequency (MAF) obtained from the 1000 Genomes Project.

### Association studies revealed different risk haplotypes in Swedish breast cancer cases and controls

The 11 variants were used to assess whether the two families’ haplotype was associated with breast cancer risk. We first performed a pilot study using the 31 linked familial cases included in the mutation screen and 95 healthy controls and found one rare risk-associated haplotype (estimated frequency 7.7% in cases and 0.3% in controls, odds ratio (OR) = 26.5, *p* = 0.0005). Although the haplotype was not the same as in the two original families, the existence of a risk haplotype among the Swedish families suggested a Swedish founder mutation at this locus.

In the next larger association study, 9 of 11 SNPs were successfully genotyped in 800 breast cancer cases and 1,750 healthy controls. There was no risk haplotype found by comparing all cases to all controls. However, one rare (3%) risk haplotype was suggested (*p* = 0.03) when the analysis included only the 496 familial breast cancer cases *versus* all 1,750 controls. This result weakly supported the hypothesis of a risk factor in the region, although the haplotype was still not the same as that seen in the two original families.

### Association studies verified the risk haplotypes and identified new candidates in European breast cancer cases and controls

To search for additional support for the region and for the two families’ haplotype, we selected SNPs from an extended region around these 11 markers to be included in a custom genotyping array project (iCOGS) of BCAC (http://bcac.ccge.medschl.cam.ac.uk/). In total 29 SNPs in the region from rs9343798 to rs7768535 were successfully genotyped in 100,165 BCAC samples (including 50,009 cases and 50,156 controls) as well as in the two original families (6006 and 6043) to search for a confirmation of the hypothesized risk haplotype identified in these two families.

The familial 29-SNP risk haplotype in the respective families was identical except for two markers (Table 2). After excluding studies of Asian women, BCAC data from 44,214 cases *vs*. 43,532 controls was first analyzed as a whole for a haplotype using the 29 markers, and next the following sub-analyses were studied, postmenopausal (age > 60, 16,878 cases), early onset (age < 50, 12,305 cases), estrogen receptor (ER) positive (26,546 cases) and ER negative (7,194 cases). Two risk haplotypes were found for all BCAC, one (I) same as in family 6006 (Table 2). The 6006 haplotype (I) was also associated with risk of breast cancer before age 50. The second haplotype (II) was associated with risk of ER-positive disease. A third haplotype (III) was associated with risk of ER-positive and late-onset (after age 60) breast cancer, and a fourth haplotype (IV) was suggested in ER-positive disease only (Table 2). Thus, the sub-analysis of ER-positive breast cancer using BCAC data identified three different haplotypes (Table 2).

**Table 2:** Candidate risk haplotypes identified in BCAC and three Swedish studies in comparison to the familial haplotypes.

			Candidate risk haplotypes									
Frequency			5.1%	5.0%	8.3%	9.0%	1.7%	1.7%	1.3%	21.3%	1.7%	5.6%
OR			1.06	1.09	1.04	1.04	1.11	1.12	1.13	1.19	1.93	1.24
*p*-value			0.03	0.02	0.04	0.04	0.05	0.02	0.02	0.01	0.02	0.006
SNP	6006	6043	BCAC I	BCAC <50 y I	BCAC II	BCAC ER+ II	BCAC >60 y III	BCAC ER+ III	BCAC ER+ IV	SASBAC	KARBAC	pKARMA >60 y
rs9343798	G	G	G	G	A	A	A	A	A	G	A	G
rs7760429	G	G	G	G	G	G	G	G	A	G	A	G
rs9350774	G	G	G	G	A	A	A	A	A	G	A	G
rs9448560	G	G	G	G	G	G	G	G	G	G	G	A
rs1507152	G	G	G	G	G	G	G	G	G	G	A	A
rs9361440	A	A	A	A	C	C	A	A	A	A	A	A
rs9343824	G	G	G	G	A	A	A	A	G	G	A	A
rs6454084	G	G	G	G	G	G	G	G	G	G	G	A
rs955765	G	G	G	G	G	G	G	G	G	G	A	G
rs9352664	A	A	A	A	C	C	C	C	A	A	C	C
rs9361459	A	A	A	A	A	A	G	G	A	A	G	G
rs9448595	G	G	G	G	G	G	G	G	G	G	G	G
rs2275290	G	G	G	G	G	G	A	A	G	G	A	G
rs10943606	C	C	C	C	C	C	C	C	C	C	A	C
rs9350797	A	A	A	A	A	A	G	G	A	A	G	A
rs10943611	A	A	A	A	G	G	A	A	A	A	A	A
rs1415862	A	A	A	A	A	A	A	A	A	A	A	G
rs1415863	G	G	G	G	G	G	G	G	G	G	G	A
rs12208915	G	G	G	G	G	G	G	G	G	G	A	G
rs9448607	A	A	A	A	G	G	G	G	A	A	G	G
rs11754374	C	C	C	C	C	C	A	A	C	C	A	C
rs10455120	A	C	A	A	A	A	A	A	A	C	A	A
rs12197385	C	A	C	C	C	C	C	C	C	A	C	C
rs1415310	A	A	A	A	G	G	G	G	A	A	G	G
rs9443645	A	A	A	A	G	G	G	G	A	A	G	G
rs12208017	A	A	A	A	A	A	C	C	A	A	C	A
rs9361491	G	G	G	G	A	A	A	A	G	G	A	G
rs6454096	G	G	G	G	G	G	A	A	G	G	A	G
rs7768535	G	G	G	G	G	G	A	A	G	G	A	G

The two families are different on markers rs10455120 and rs12197385. Sub-analyses on postmenopausal (>60 y), early onset (<50 y), ER positive (ER+) and negative (ER-) cases were performed in all four studies. Estimated haplotype frequency in the control group, OR and association p-value was shown for each risk haplotype. Four risk haplotypes revealed in the BCAC study were referred as I, II, III and IV.

The Swedish BCAC studies are the most likely to have included patients with a Swedish founder mutation with risk haplotypes around it. In the SASBAC study (1,163 cases *vs*. 1,378 controls) recruiting postmenopausal cases, the same haplotype as in family 6043 was associated with breast cancer risk (Table 2). In the KARBAC study (722 cases *vs*. 662 controls) with a predominance of familial breast cancer cases, it was suggested a different susceptibility haplotype, and also a higher associated risk compared to the other risk haplotypes (Table 2). Finally, the third Swedish study pKARMA (4,553 cases *vs*. 5,537 controls), with consecutive cases from a mammography cohort, suggested yet another risk-associated haplotype (Table 2). The result altogether further supported the hypothesis of a breast cancer risk locus on chromosome 6q14.

The risk-associated haplotype identified using KARBAC was not studied further since the 29 SNPs were only genotyped in non-related individuals and relatives from the two original families. We hypothesized that the distribution of risk haplotypes of different sizes over a locus could reflect one or several risk alleles. Thus, we performed a haplotype analysis in KARBAC on sliding windows over the 29 SNPs. The result suggested the existence of one risk allele in the *PHIP* gene (Supplementary Figure 1a). Sliding-window haplotype analyses were also performed for the whole BCAC and the other Swedish studies SASBAC and pKARMA (Table 2 and Supplementary Figure 1). The BCAC risk haplotype I (in BCAC and in BCAC < 50), same as the one in family 6006, showed a similar result as in KARBAC, one risk allele in the *PHIP* gene (Supplementary Figure 1b). The almost same risk haplotype, originally identified in family 6043 and observed using SASBAC data, instead suggested involvement of one locus in the 5’-part of the *PHIP* gene as well as another locus proximal to *PHIP* (Supplementary Figure 1c). The same analysis for the other haplotypes BCAC (II, III and IV) and pKARMA suggested three possible loci (BCAC III and pKARMA), one proximal to, one in, and one distal to *PHIP*. The BCAC II and IV haplotypes both focus on the suggested risk allele distal to *PHIP* (data not shown).

We had previously tested for mutations in the exonic regions over the whole haplotype in the two families without finding any candidate mutation. A targeted genome sequencing approach was subsequently applied to a 2.8 Mb region in the two original families (from rs9447790 to rs2655685) and the lack of clear pathogenic mutations was confirmed. The two families shared many markers in the region of the haplotype. It was not possible to define the pathogenic mutation in either of the two haplotype regions (Supplementary Table 1).

## DISCUSSION

The study was based on the early finding that two unrelated families shared one haplotype for a risk locus on chromosome 6q. It was also hypothesized, based on the family history and the design used (linkage analysis), that they shared a mutation in a rare, high-risk breast cancer gene. In the end, it was clear that they did not share an identical haplotype. Family 6006 had one identical to a risk haplotype in BCAC, in particular for those with early onset and family 6043 was similar to one quite prevalent (frequency 21%) haplotype in Swedish postmenopausal cases (SASBAC) (Table 2). It was also suggested that the families had different mutations, 6006 within the *PHIP* gene and 6043 one or two proximal and distal to the *PHIP* gene (Supplementary Figure 1).

This risk locus on 6q was first the result from two different linkage studies in familial breast cancer and chosen for further study, not because of a statistically significant LOD score, but rather that the locus was suggested from two separate studies, and that two unrelated families seemed to share a unique haplotype at this locus. The lack of statistically significant loci in linkage analysis in breast cancer is often seen and perhaps results from a complex inheritance pattern in genetically unsolved familial breast cancer. Sanger sequencing excluded an exome-based mutation at the locus in the two families. However, early small association studies gave support for a familial risk haplotype in the region. This first association study performed using only 31 samples, selected because of possible linkage to the locus, suggested a clear association to this locus (*p* = 0.0005). However, the evidence was much weaker when sample sizes increased (*p* = 0.03). All the time the focus was on familial samples, which might explain the difficulties to identify the two original families’ haplotypes since they are not over-represented among familial samples (Table 2). It was not until it was possible to study also sporadic breast cancer samples within BCAC that evidence was found for the families’ haplotypes to act as risk alleles in breast cancer. It was most clear in SASBAC with postmenopausal cases.

The target for the risk association is between our original borders D6S1625 - rs2050660 and in this region there are several genes such as *HTR1B, MEI4, IRAK1BP1, PHIP, HMGN3, LCA5* and *SH3BGRL2* as well as several different RNAs. The *HTR1B* gene has been suggested to be involved in breast cancer progression [27] and the *HMGN3* gene has been published to be upregulated in breast cancer cell lines [28]. None is currently known to be associated with breast cancer risk. We were mostly interested in the region of the *PHIP* gene and have focused our investigation around it because of the shared haplotype in the two families hypothesized as a risk haplotype over this region. In fact, the *PHIP* gene was suggested to be target of the risk allele in KARBAC with familial cases and BCAC early onset cases (Supplementary Figure 1a and 1b). It has been reported that pleckstrin homology domain interacting protein (PHIP) promotes tumor metastasis through Akt activation in murine melanoma [29]. Forward genetic screen in mice with malignant peripheral nerve sheath tumor also identified the *PHIP* gene as a common integration site, indicating its role as a candidate driver gene in tumorigenesis [30]. The *PHIP* gene has been associated with melanoma progression, and overexpression has been suggested to serve as an independent adverse predictor of survival in melanoma [29]. To our knowledge there is not yet any study on *PHIP* gene in relation to breast cancer prognosis or risk. We did find one unique missense mutation c.A3733G (p.I1245V) in one family (6082). This mutation did not segregate in affected members in this family. The other two missense variants in this gene, rs11547228 (p.T874I) and rs7747479 (p.G663V), did not show any increased frequency in the 48 breast cancers subjected to targeted sequencing compared to that in a general population (1000 Genomes Project). Although none of the 29 SNPs shows statistically significant evidence of being an eQTL in breast mammary tissue in GTEx [31], ENCODE project has revealed that this locus contains multiple regulatory elements including promoters, enhancers, transcription factor binding sites, open chromatin regions, DNaseI hypersensitivity sites, etc [32]. It is possible that an involvement of the gene is related to its expression level.

Linkage analysis typically has been used to find high-penetrance disease [4, 5] while association studies typically reveal low-penetrance risk loci [23]. Our studies were first set up to detect a highly penetrant breast cancer susceptibility gene using familial breast cancer. This approach was not successful (negative Sanger sequencing in all genes in the region) and it was clear that association studies might be more powerful than linkage studies (statistically significant support for a risk haplotype in our early studies). Thus, the candidate region was tested using association analysis within the iCOGS project in BCAC. The finding of several low risk haplotypes in this risk locus demonstrated also different risks, with an estimated OR of 1.1 for the common and typical low-risk haplotypes in SASBAC and BCAC, while the moderate-risk haplotype in the familial cohort KARBAC had an OR of almost 2 and was rare. Low-risk loci often do not involve exonic mutations but are frequently distributed over gene-free regions [23], which makes it more difficult to clearly define the disease-causing mutation. Thus, no clear pathogenic mutation was identified on the haplotype with too many candidate mutations over the region.

The project has been carried out over a long period and the methods used were appropriate at the time (Supplementary Figure 2). The first linkage analysis was set up in 1998, completed in 2002 and chose the best technique and markers available at the time [25]. The second linkage study was completed much later and used more informative markers [26]. Still, in spite of the overall lack of positive LOD scores, both studies suggested two loci (on chromosomes 3 and 6) with weak evidence, but one was of particular interest because of the two families with the shared haplotype and early findings of same gene expression profiles in tumors (unpublished data).

Linkage analysis has shown its limitations to define novel loci for a heterogeneous and complex disease, such as breast cancer [7-14, 25, 26]. Association studies will typically provide evidence for low-risk alleles and the clinical impact of these alleles are yet to be determined [23]. Today next-generation sequencing presents new opportunities to use direct sequencing of patients with familial breast cancer [33-36]. We have used several strategies to study a small region on chromosome 6q14 and found support for a breast cancer susceptibility locus, related to the *PHIP* gene, with low and moderate risk profiles. Further studies of patients with the identified haplotypes and their tumors will be necessary before it is possible to estimate the importance of this locus. Haplotype analysis in populations with a homogenous genetic background will be valuable to further define this locus.

## MATERIALS AND METHODS

### Families for linkage studies and cases for association studies

Non-BRCA1/2 breast cancer families were recruited through the Cancer Counseling Clinic at the Karolinska University Hospital, Solna, Sweden. The first linkage study involved 272 members from 96 non-BRCA1/2 breast cancer families, including 232 breast cancer patients (age of diagnosis 30-72 years, mean 54.7 years). These families were selected if two or more first- or second-degree relatives were affected with only female breast cancer, and if the proband had counseled a two to four times increased risk for the disease compared to the normal population, no age limitation required. Twenty of these families have two affected cases, 42 families have three cases, 24 families have four cases, eight families have five cases and two families have six cases, with an average of 3.2 cases per family. Nine of the families were breast-ovarian cancer families. No other types of cancer were presented in this set of families.

The second linkage study was based on the cohort of 14 large hereditary non-BRCA1/2 breast cancer families with also other cancers in the family [26]. DNA was available from 96 family members, including 50 affected by breast cancer. Linkage analysis tested patients were grouped as breast cancer families where only breast cancer cases were coded as affected, and also using cancer syndrome criteria wherein patients having breast, cervical, endometrial, colorectal, prostate or any other cancers were coded as affected.

The association studies used 300 familial cases identified and recruited as described above and 500 consecutive breast cancer cases from a breast cancer clinic at Södersjukhuset in Stockholm [37]. The consecutive cases were recruited at diagnosis and can be categorized as familial or sporadic cases if a family history of cancer was available. Blood samples were obtained from affected family members with informed consent. Blood donors from the same hospital and cancer-free spouses served as controls. Genomic DNAs were extracted using standard phenol extraction procedures.

The study was undertaken in accordance with the Swedish legislation of ethical permission (2003:460) and according to the decision in the Stockholm regional ethical committee in Stockholm (Dnr: 1992/207. 1997/205, 1998/232, 2000/291).

### Linkage analysis in 96 breast and breast-ovarian cancer families

Genotyping was carried out using Linkage Mapping Set v2.0- MD10 (Applied Biosystems). 400 fluorescent-labeled microsatellite markers covered the whole genome with an average resolution of 10 cM. The average heterozygosity of these markers was 0.76 in our sample set. The amplified fragments were separated on an ABI 377 automated sequencer (Applied Biosystems) together with internal size standard. Electrophoretic data were analyzed using GeneScan (v3.1) and Genotyper (v2.0) softwares (Applied Biosystems).

Genotyping data were first checked for consistency by using PedCheck [38]. Homozygosity tests were performed to further check marker quality and genotyping errors. Model-dependent multipoint LOD scores were generated using GeneHunter v2.1 [39] complemented by LINKMAP of FASTLINK [40-42]. Two-point LOD scores were performed using the MLINK of the FASTLINK package because multipoint LOD scores are always affected by flanking markers and marker density. We assigned an autosomal dominant inheritance model in the linkage analysis, with only female breast cancer as the affected phenotype. The gene frequency was set to 0.003. Age-dependent penetrance for carriers was applied. The penetrance was set to 0.01 for unaffected males, 0.20 for unaffected females below 40 years old, 0.40 for unaffected females between 40-65 years old and 0.60 for unaffected females above 65 years old. Marker allele frequencies were estimated by PedCheck from individuals in each pedigree. Published map distances from Marshfield [43] were used in the analysis. The overall LOD scores were maximized to allow for heterogeneity (HLOD). At each locus a maximum HLOD and a corresponding estimate of the proportion of families linked were obtained.

### Linkage analysis in 14 large breast cancer families with other cancers

The second linkage analysis was carried out in 14 large breast cancer families with other cancer types using a previously published approach [26] with modifications. We defined affected status as: 1) breast cancer only; 2) breast cancer and other cancers in two separate analyses, in order to search for additional evidence of risk loci associated with breast cancer.

### Fine-mapping of the chromosome 6q14 locus following linkage analyses

Markers were selected throughout the chromosome 6q14 region revealed by the linkage analyses for fine-mapping in additional breast cancer families. Primers for the markers were obtained from dbSTS (http://www.ncbi.nlm.nih.gov/projects/dbSTS/) and were labeled with 6-Fam Fluorescein modification at the 5’ end of forward primers. The amplified markers were analyzed on an ABI Prism 377, 3130xL or 3500xL Genetic Analyzer (Applied Biosystems). Softwares GeneScan (v3.1R), GeneMapper (v3.7 or 4.1) and GenoTyper (v2.0) was used to analyze the peaks.

### Sanger sequencing of linked familial breast cancer samples

Genomic DNA from one affected member from each of the 31 families with positive LOD scores for the linked region, including families 6006 and 6043, was PCR-amplified and sequenced for the six genes within this region (*HTR1B*, *IRAK1BP1*, *PHIP*, *HMGN3*, *C6ORF152* and *SH3BGRL2*) for all exons including exon/intron boundaries, 5’ and 3’ UTR sequences and putative promoter regions. All genes were successfully amplified and sequenced. RT-PCR analysis was carried out using mRNA from EBV-transformed lymphocytes from two cases in each of the families 6006 and 6043 to detect aberrant splicing patterns or large insertions / deletions.

### Copy number analysis by array-CGH

A custom array-CGH was designed for exon specific analysis of deletions and duplications in the chromosome 6 region (77462539-80350534). Agilent Technologies Suredesign was used to design a targeted 4x180K array (Oxford Gene Technologies, Oxfordshire, UK). Experiments were performed at the Department of Clinical Genetics at Karolinska University Hospital, Stockholm, Sweden according to the manufacturer’s protocol. Slides were scanned using the Agilent Microarray Scanner (G2505C, Agilent technologies, USA). Raw data were normalized using Feature Extraction Software (10.7.3.1, Agilent Technologies, USA), and log2 ratios were calculated by dividing the normalized intensity in the sample by the mean intensity across the reference sample. The log2 ratios were plotted and segmented by circular binary segmentation in the CytoSure Interpret software (Oxford Gene Technology, Oxfordshire, UK). Oligonucleotide probe positions were annotated to Human Genome Assembly hg19.

### Association study using 9 SNPs from Sanger sequencing

Nine variants (shared sequence variants in family 6006 and 6043) used for the association study in 800 cases and 1750 controls were rs2275291, rs2275290, rs1984195, rs9443632, rs10455356, rs9350797, rs7742431, rs1890229 and rs11752126 genotyped by DeCODE, Iceland.

### Genotyping of BCAC samples

Genotyping within BCAC and iCOGS used a custom Illumina array with 211,155 SNPs. Genotyping, allele calling, quality control and principal components analysis for the iCOGS study are described in detail in Michailidou et al. [23]. In total 29 SNPs were successfully genotyped for the 6q region. Haplotype association analysis was performed on the whole BCAC study as well as on three Swedish subpopulations independently. Haplotype frequency was estimated for all but Asian samples and for all 29 markers. P-values were calculated by Plink v.1.07 [44].

### Capture sequencing

Capture sequencing of 48 familial breast cancer patients was performed by Axeq Technologies, USA using a SureSelect target enrichment process followed by 100 bp paired-end sequencing on an Illumina HiSeq2000 sequencer. After sequencing, bioinformatics analysis of the FASTQ files included alignment of sequence reads to the reference human genome (GRCh37/hg19) using BWA and SAMTools, applying GATK [45-47] base quality score recalibration, indel realignment, duplicate removal, variant calling and annotation (dbSNP and 1000 Genome Project).

## SUPPLEMENTARY MATERIALS FIGURES AND TABLE





## Abbreviations

SNP: single nucleotide polymorphism
NPL: non-parametric linkage
LOD: logarithm of odds
HLOD: heterogeneity LOD
OR: odds ratio
MAF: minor allele frequency
Mb: megabase
bp: basepair
cM: centi Morgan
