# Supplementary material for: PHIP - a novel candidate breast cancer susceptibility locus on 6q14.1
Source: Oncotarget. 2017 Oct 12;8(61):102769–82. doi: 10.18632/oncotarget.21800 (PMC5732689; doi:10.18632/oncotarget.21800)
Supplement: Supplementary file 1 [file oncotarget-08-102769-s001.pdf]

PHIP - a novel candidate breast cancer susceptibility locus on 6q14.1

SUPPLEMENTARY MATERIALS

Supplemental Note: Acknowledgements and funding of individual BCAC studies

The Australian Breast Cancer Family Study (ABCFS) thanks Maggie Angelakos, Judi Maskiell and Gillian Dite. The ABCFS was supported by grant UM1 CA164920 from the National Cancer Institute (USA). The content of this manuscript does not necessarily reflect the views or policies of the National Cancer Institute or any of the collaborating centers in the Breast Cancer Family Registry (BCFR), nor does mention of trade names, commercial products, or organizations imply endorsement by the USA Government or the BCFR. The ABCFS was also supported by the National Health and Medical Research Council of Australia, the New South Wales Cancer Council, the Victorian Health Promotion Foundation (Australia) and the Victorian Breast Cancer Research Consortium. J.L.H. is a National Health and Medical Research Council (NHMRC) Senior Principal Research Fellow. M.C.S. is a NHMRC Senior Research Fellow.

The Amsterdam Breast Cancer Study (ABCS) thanks Blood bank Sanquin, The Netherlands. The ABCS study was supported by the Dutch Cancer Society [grants NKI 2007-3839; 2009 4363].

The work of the Bavarian Breast Cancer Cases and Controls (BBCC) was partly funded by ELAN-Fond of the University Hospital of Erlangen.

The British Breast Cancer Study (BBCS) thanks Eileen Williams, Elaine Ryder-Mills and Kara Sargus. The BBCS is funded by Cancer Research UK and Breast Cancer Now and acknowledges NHS funding to the NIHR Biomedical Research Centre and the National Cancer Research Network (NCRN).

The Breast Cancer in Galway Genetic Study (BIGGS) thanks Niall McInerney, Gabrielle Colleran, Andrew Rowan and Angela Jones. ES is supported by NIHR Comprehensive Biomedical Research Centre, Guy's & St. Thomas' NHS Foundation Trust in partnership with King's College London, United Kingdom. IT is supported by the Oxford Biomedical Research Centre.

The Breast Cancer Study of the University of Heidelberg (BSUCH) thanks Peter Bugert and Medical Faculty Mannheim. The BSUCH study was supported by the Dietmar-Hopp Foundation, the Helmholtz Society and the German Cancer Research Center (DKFZ).

The CECILE Breast Cancer Study was supported by Fondation de France, Institut National du Cancer (INCa), Ligue Nationale contre le Cancer, Agence Nationale de Sécurité Sanitaire, de l'Alimentation, de l'Environnement et du Travail (ANSES), Agence Nationale de la Recherche (ANR).

The Copenhagen General Population Study (CGPS) thanks the staff and participants of the Copenhagen General Population Study. For the excellent technical assistance: Dorthe Uldall Andersen, Maria Birna Arnadottir, Anne Bank and Dorthe Kjeldgård Hansen. The Danish Cancer Biobank is acknowledged for providing infrastructure for the collection of blood samples for the cases. The CGPS was supported by the Chief Physician Johan Boserup and Lise Boserup Fund, the Danish Medical Research Council, and Herlev and Gentofte Hospital.

The Spanish National Cancer Centre Breast Cancer Study (CNIO-BCS) thanks Guillermo Pita, Charo Alonso, Nuria Álvarez, Pilar Zamora, Primitiva Menendez and the Human Genotyping-CEGEN Unit (CNIO). The CNIO-BCS was supported by the Instituto de Salud Carlos III, the Red Temática de Investigación Cooperativa en Cáncer and grants from the Asociación Española Contra el Cáncer and the Fondo de Investigación Sanitario (PI11/00923 and PI12/00070).

The California Teachers Study (CTS) Steering Committee includes Leslie Bernstein, Susan Neuhausen, James Lacey, Sophia Wang, Huiyan Ma, and Jessica Clague DeHart at the Beckman Research Institute of City of Hope, Dennis Deapen, Rich Pinder, and Eunjung Lee at the University of Southern California, Pam Horn-Ross, Peggy Reynolds, Christina Clarke Dur and David Nelson at the Cancer Prevention Institute of California, Hoda Anton-Culver, Argyrios Ziogas, and Hannah Park at the University of California Irvine, and Fred Schumacher at Case Western University. The CTS was initially supported by the California Breast Cancer Act of 1993 and the California Breast Cancer Research Fund (contract 97-10500) and is currently funded through the National Institutes of Health (R01 CA77398, UM1 CA164917, and U01 CA199277). Collection of cancer incidence data was supported by the California Department of Public Health as part of the statewide cancer reporting program mandated by California Health and Safety Code Section 103885. HAC receives support from the Lon V Smith Foundation (LVS39420).

The ESTHER Breast Cancer thanks Hartwig Ziegler, Sonja Wolf, Volker Hermann, Christa Stegmaier and Katja Butterbach. The ESTHER study was supported by a grant from the Baden Württemberg Ministry of Science, Research and Arts. Additional cases were recruited in the context of the VERDI study, which was supported by a grant from the German Cancer Aid (Deutsche Krebshilfe).

The German Consortium of Hereditary Breast and Ovarian Cancer (GC-HBOC) thanks Stefanie Engert, Heide Hellebrand, Sandra Kröber and LIFE - Leipzig Research Centre for Civilization Diseases (Markus Loeffler, Joachim Thiery, Matthias Nüchter, Ronny Baber). The GC-HBOC is supported by the German Cancer Aid (grant no 110837, coordinator: Rita K. Schmutzler, Cologne). This work was also funded by the European Regional Development Fund and Free State of Saxony, Germany (LIFE - Leipzig Research Centre for Civilization Diseases, project numbers 713-241202, 713-241202, 14505/2470, 14575/2470).

The Gene Environment Interaction and Breast Cancer in Germany (GENICA) Network: Dr. Margarete Fischer-Bosch-Institute of Clinical Pharmacology, Stuttgart, and University of Tübingen, Germany [HB, Wing-Yee Lo, Christina Justenhoven], German Cancer Consortium (DKTK) and German Cancer Research Center (DKFZ) [HB], Department of Internal Medicine, Evangelische Kliniken Bonn gGmbH, Johanniter Krankenhaus, Bonn, Germany [Yon-Dschun Ko, Christian Baisch], Institute of Pathology, University of Bonn, Germany [Hans-Peter Fischer], Molecular Genetics of Breast Cancer, Deutsches Krebsforschungszentrum (DKFZ), Heidelberg, Germany [Ute Hamann], Institute for Prevention and Occupational Medicine of the German Social Accident Insurance, Institute of the Ruhr University Bochum (IPA), Bochum, Germany [TB, Beate Pesch, Sylvia Rabstein, Anne Lotz]; and Institute of Occupational Medicine and Maritime Medicine, University Medical Center Hamburg-Eppendorf, Germany [Volker Harth]. The GENICA was funded by the Federal Ministry of Education and Research (BMBF) Germany grants 01KW9975/5, 01KW9976/8, 01KW9977/0 and 01KW0114, the Robert Bosch Foundation, Stuttgart, Deutsches Krebsforschungszentrum (DKFZ), Heidelberg, the Institute for Prevention and Occupational Medicine of the German Social Accident Insurance, Institute of the Ruhr University Bochum (IPA), Bochum, as well as the Department of Internal Medicine, Evangelische Kliniken Bonn gGmbH, Johanniter Krankenhaus, Bonn, Germany.

The Helsinki Breast Cancer Study (HEBCS) thanks Sofia Khan, Johanna Kiiski, Kristiina Aittomäki, Rainer Fagerholm, Kirsimari Aaltonen, Karl von Smitten and Irja Erkkilä. The HEBCS was financially supported by the Helsinki University Central Hospital Research Fund, Academy of Finland (266528), the Finnish Cancer Society, The Nordic Cancer Union and the Sigrid Juselius Foundation.

The Hannover-Minsk Breast Cancer Study (HMBCS) thanks Peter Hillemanns, Hans Christiansen and Johann H. Karstens. The HMBCS was supported by a grant from the Friends of Hannover Medical School and by the Rudolf Bartling Foundation.

Financial support for Karolinska Breast Cancer Study (KARBAC) was provided through the regional agreement on medical training and clinical research (ALF) between Stockholm County Council and Karolinska Institutet, the Swedish Cancer Society, The Gustav V Jubilee foundation and Bert von Kantzows foundation.

The Kuopio Breast Cancer Project (KBCP) thanks Eija Myöhänen and Helena Kemiläinen. The KBCP was financially supported by the special Government Funding (EVO) of Kuopio University Hospital grants, Cancer Fund of North Savo, the Finnish Cancer Organizations, and by the strategic funding of the University of Eastern Finland.

The Kathleen Cuningham Foundation Consortium for research into Familial Breast Cancer/Australian Ovarian Cancer

Study (kConFab/AOCS) wish to thank Heather Thorne, Eveline Niedermayr, all the kConFab research nurses and staff, the heads and staff of the Family Cancer Clinics, and the Clinical Follow Up Study (which has received funding from the NHMRC, the National Breast Cancer Foundation, Cancer Australia, and the National Institute of Health (USA)) for their contributions to this resource, and the many families who contribute to kConFab. kConFab is supported by a grant from the National Breast Cancer Foundation, and previously by the National Health and Medical Research Council (NHMRC), the Queensland Cancer Fund, the Cancer Councils of New South Wales, Victoria, Tasmania and South Australia, and the Cancer Foundation of Western Australia. Financial support for the AOCS was provided by the United States Army Medical Research and Materiel Command [DAMD17-01-1-0729], Cancer Council Victoria, Queensland Cancer Fund, Cancer Council New South Wales, Cancer Council South Australia, The Cancer Foundation of Western Australia, Cancer Council Tasmania and the National Health and Medical Research Council of Australia (NHMRC; 400413, 400281, 199600). G.C.T. and P.W. are supported by the NHMRC. RB was a Cancer Institute NSW Clinical Research Fellow.

The Leuven Multidisciplinary Breast Centre (LMBC) thanks Gilian Peuteman, Thomas Van Brussel, EvyVanderheyden and Kathleen Corthouts. LMBC is supported by the "Stichting tegen Kanker". Diether Lambrechts is supported by the FWO.

The Mammary Carcinoma Risk Factor Investigation (MARIE) thanks Petra Seibold, Judith Heinz, Nadia Obi, Alina Vrieling, Sabine Behrens, Ursula Eilber, Muhabbet Celik, Til Olchers and Stefan Nickels. The MARIE study was supported by the Deutsche Krebshilfe e.V. [70-2892-BR I, 106332, 108253, 108419, 110826, 110828], the Hamburg Cancer Society, the German Cancer Research Center (DKFZ) and the Federal Ministry of Education and Research (BMBF) Germany [01KH0402].

The Milan Breast Cancer Study Group (MBCSG) thanks Siranoush Manoukian, Bernard Peissel, Jacopo Azzollini, Daniela Zaffaroni and Lidia Pezzani of the Fondazione IRCCS Istituto Nazionale dei Tumori (INT); Paolo Peterlongo of IFOM, the FIRC Institute of Molecular Oncology; Irene Feroce of the Istituto Europeo di Oncologia (IEO) and the personnel of the Cogentech Cancer Genetic Test Laboratory. The MBCSG is supported by grants from the Italian Association for Cancer Research (AIRC) and by funds from the Italian citizens who allocated the 5/1000 share of their tax payment in support of the Fondazione IRCCS Istituto Nazionale Tumori, according to Italian laws (INT-Institutional strategic projects "5x1000").

The Mayo Clinic Breast Cancer Study (MCBCS) was supported by the NIH grants CA192393, CA116167, CA176785 an NIH Specialized Program of Research Excellence (SPORE) in Breast Cancer [CA116201], and the Breast Cancer Research Foundation and a generous gift from the David F. and Margaret T. Grohne Family Foundation.

The Melbourne Collaborative Cohort Study (MCCS) cohort recruitment was funded by VicHealth and Cancer Council Victoria. The MCCS was further supported by Australian NHMRC grants 209057, 251553 and 504711 and by infrastructure provided by Cancer Council Victoria. Cases and their vital status were ascertained through the Victorian Cancer Registry (VCR) and the Australian Institute of Health and Welfare (AIHW), including the National Death Index and the Australian Cancer Database.

The Multi-ethnic Cohort (MEC) was support by NIH grants CA63464, CA54281, CA098758, CA132839 and CA164973.

MTLGEBCS would like to thank Martine Tranchant (CHU de Québec Research Center), Marie-France Valois, Annie Turgeon and Lea Heguy (McGill University Health Center, Royal Victoria Hospital; McGill University) for DNA extraction, sample management and skillful technical assistance. J.S. is Chairholder of the Canada Research Chair in Oncogenetics. The work of MTLGEBCS was supported by the Quebec Breast Cancer Foundation, the Canadian Institutes of Health Research for the "CIHR Team in Familial Risks of Breast Cancer" program – grant # CRN-87521 and the Ministry of Economic Development, Innovation and Export Trade – grant # PSR-SIIRI-701.

The Norwegian Breast Cancer Study (NBCS) Collaborators: Prof. Em. Anne-Lise Børresen-Dale, PhD (Department of Cancer Genetics, Institute for Cancer Research, Oslo University Hospital Radiumhospitalet, Oslo, Norway), Dr. Kristine K. Sahlberg, PhD (Department of Research, Vestre Viken Hospital, Drammen, Norway and Department of Cancer Genetics, Institute for Cancer Research, Oslo University Hospital Radiumhospitalet, Oslo, Norway), Dr. Lars Ottestad, MD (Department of Cancer Genetics, Institute for Cancer Research, Oslo University Hospital Radiumhospitalet, Oslo, Norway), Prof. Em. Rolf Kåresen, MD (Institute of Clinical Medicine, Faculty of Medicine, University of Oslo, Oslo, Norway and Department of Breast- and Endocrine Surgery, Division of Surgery, Cancer and Transplantation Medicine, Oslo University Hospital Ullevål, Oslo, Norway), Dr. Anita Langerød, PhD (Department of Cancer Genetics, Institute for Cancer Research, Oslo University

Hospital Radiumhospitalet, Oslo, Norway), Dr. Ellen Schlichting, MD (Section for breast- and endocrine surgery, Department of oncology, Oslo university hospital), Dr. Marit Muri Holmen, MD (Department of Radiology and Nuclear Medicine, Oslo University Hospital, Oslo, Norway), Prof. Toril Sauer, MD (Institute of Clinical Medicine, Faculty of Medicine, University of Oslo, Oslo, Norway and Department of Pathology at Akershus University hospital, Lørenskog, Norway), Dr. Vilde Haakensen, MD (Department of Cancer Genetics, Institute for Cancer Research, Oslo University Hospital Radiumhospitalet, Oslo, Norway), Dr. Olav Engebråten, MD (Institute of Clinical Medicine, Faculty of Medicine, University of Oslo, Oslo, Norway, Department of Tumor Biology, Institute for Cancer Research, Oslo University Hospital Radiumhospitalet, Oslo, Norway and Department of Oncology, Division of Surgery and Cancer and Transplantation Medicine, Oslo University Hospital Radiumhospitalet, Oslo, Norway), Prof. Bjørn Naume, MD (Department of Oncology, Oslo University Hospital, Oslo, Norway and Institute of Clinical Medicine, Faculty of Medicine, University of Oslo, Oslo, Norway), Dr. Alexander Fosså, MD (National Advisory Unit on Late Effects after Cancer Treatment, Department of Oncology, Oslo University Hospital, Oslo, Norway and Department of Oncology, Oslo University Hospital Ullevål, Oslo, Norway), Dr. Cecile E. Kiserud, MD (National Advisory Unit on Late Effects after Cancer Treatment, Department of Oncology, Oslo University Hospital, Oslo, Norway and Department of Oncology, Oslo University Hospital Ullevål, Oslo, Norway), Dr. Kristin V. Reinertsen, MD (National Advisory Unit on Late Effects after Cancer Treatment, Department of Oncology, Oslo University Hospital, Oslo, Norway and Department of Oncology, Oslo University Hospital Ullevål, Oslo, Norway), Assoc. Prof. Åslaug Helland, MD (Department of Cancer Genetics, Institute for Cancer Research, Oslo University Hospital Radiumhospitalet, Oslo, Norway), Dr. Margit Riis, MD (Department of Breast- and Endocrine Surgery, Division of Surgery, Cancer and Transplantation Medicine, Oslo University Hospital Ullevål, Oslo, Norway), Prof. Jürgen Geisler, MD (Institute of Clinical Medicine, Faculty of Medicine, University of Oslo, Oslo, Norway and Department of Oncology, Akershus University Hospital, Oslo, Norway), Prof. Per Eystein Lønning, MD (Section of Oncology, Institute of Medicine, University of Bergen and Department of Oncology, Haukeland University Hospital, Bergen, Norway), Grethe I. Grenaker Alnæs, M.Sc. (Department of Cancer Genetics, Institute for Cancer Research, Oslo University Hospital Radiumhospitalet, Oslo, Norway) and OSBREAC (Oslo Breast Cancer Research Consortium, Oslo University Hospital, Oslo, Norway). The NBCS has been supported by the Research Council of Norway grant 193387/V50 (to A-L Børresen-Dale and V.N. Kristensen) and grant 193387/H10 (to A-L Børresen-Dale and V.N. Kristensen), South Eastern Norway Health Authority (grant 39346 to A-L Børresen-Dale and 27208 to V.N.Kristensen) and the Norwegian Cancer Society (to A-L Børresen-Dale and 419616 - 71248 - PR-2006-0282 to V.N. Kristensen). It has received funding from the K.G. Jebsen Centre for Breast Cancer Research (2012-2015).

The Nashville Breast Health Study (NBHS) thanks study participants and research staff for their contributions and commitment to this study. The NBHS was supported by NIH grant R01CA100374. Biological sample preparation was conducted the Survey and Biospecimen Shared Resource, which is supported by P30 CA68485.

The Oulu Breast Cancer Study (OBCS) thanks Arja Jukkola-Vuorinen, Mervi Grip, Saila Kauppila, Meeri Otsukka, Leena Keskitalo and Kari Mononen for their contributions to this study. The OBCS was supported by research grants from the Finnish Cancer Foundation, the Academy of Finland (grant number 250083, 122715 and Center of Excellence grant number 251314), the Finnish Cancer Foundation, the Sigrid Juselius Foundation, the University of Oulu, the University of Oulu Support Foundation and the special Governmental EVO funds for Oulu University Hospital-based research activities.

The Ontario Familial Breast Cancer Registry (OFBCR) thanks Teresa Selander and Nayana Weerasooriya. The OFBCR was supported by grant UM1 CA164920 from the National Cancer Institute (USA). The content of this manuscript does not necessarily reflect the views or policies of the National Cancer Institute or any of the collaborating centers in the Breast Cancer Family Registry (BCFR), nor does mention of trade names, commercial products, or organizations imply endorsement by the USA Government or the BCFR.

The Leiden University Medical Centre Breast Cancer Study (ORIGO) thanks E. Krol-Warmerdam, and J. Blom for patient accrual, administering questionnaires, and managing clinical information. The LUMC survival data were retrieved from the Leiden hospital-based cancer registry system (ONCDOC) with the help of Dr. J. Molenaar. The ORIGO study was supported by the Dutch Cancer Society (RUL 1997-1505) and the Biobanking and Biomolecular Resources Research Infrastructure (BBMRI-NL CP16).

The NCI Polish Breast Cancer Study (PBCS) thanks Louise Brinton, Mark Sherman, Neonila Szeszenia-Dabrowska, Beata Peplonska, Witold Zatonski, Pei Chao and Michael Stagner. The PBCS was funded by Intramural Research Funds of the National Cancer Institute, Department of Health and Human Services, USA.

The Karolinska Mammography Project for Risk Prediction of Breast Cancer - prevalent cases (pKARMA) thanks the

Swedish Medical Research Counsel. The pKARMA study was supported by Märit and Hans Rausings Initiative Against Breast Cancer.

The Rotterdam Breast Cancer Study (RBCS) thanks Petra Bos, Jannet Blom, Ellen Crepin, Elisabeth Huijskens, Anja Kromwijk-Nieuwlaat, Annette Heemskerk and the Erasmus MC Family Cancer Clinic. The RBCS was funded by the Dutch Cancer Society (DDHK 2004-3124, DDHK 2009-4318).

The Singapore and Sweden Breast Cancer Study (SASBAC) thanks the Swedish Medical Research Counsel. The SASBAC study was supported by funding from the Agency for Science, Technology and Research of Singapore (A*STAR), the US National Institute of Health (NIH) and the Susan G. Komen Breast Cancer Foundation.

The Sheffield Breast Cancer Study (SBCS) thanks Sue Higham, Helen Cramp, Dan Connley, Ian Brock, Sabapathy Balasubramanian and Malcolm W.R. Reed. The SBCS was supported by Sheffield Experimental Cancer Medicine Centre and Breast Cancer Now Tissue Bank.

The Southern Community Cohort Study (SCCS) is supported by a grant from the National Institutes of Health (R01 CA092447). Data on SCCS cancer cases used in this publication were provided by the Alabama Statewide Cancer Registry; Kentucky Cancer Registry, Lexington, KY; Tennessee Department of Health, Office of Cancer Surveillance; Florida Cancer Data System; North Carolina Central Cancer Registry, North Carolina Division of Public Health; Georgia Comprehensive Cancer Registry; Louisiana Tumor Registry; Mississippi Cancer Registry; South Carolina Central Cancer Registry; Virginia Department of Health, Virginia Cancer Registry; Arkansas Department of Health, Cancer Registry, 4815 W. Markham, Little Rock, AR 72205. The Arkansas Central Cancer Registry is fully funded by a grant from National Program of Cancer Registries, Centers for Disease Control and Prevention (CDC). Data on SCCS cancer cases from Mississippi were collected by the Mississippi Cancer Registry which participates in the National Program of Cancer Registries (NPCR) of the Centers for Disease Control and Prevention (CDC). The contents of this publication are solely the responsibility of the authors and do not necessarily represent the official views of the CDC or the Mississippi Cancer Registry.

The Study of Epidemiology and Risk factors in Cancer Heredity (SEARCH) thanks the SEARCH and EPIC teams. The SEARCH is funded by a programme grant from Cancer Research UK [C490/A10124] and supported by the UK National Institute for Health Research Biomedical Research Centre at the University of Cambridge.

The Städtisches Klinikum Karlsruhe Deutsches Krebsforschungszentrum Study (SKKDKFZS) thanks all study participants, clinicians, family doctors, researchers and technicians for their contributions and commitment to this study. The SKKDKFZS is supported by the DKFZ.

The IHCC-Szczecin Breast Cancer Study (SZBCS) thanks Ewa Putresza. The SZBCS was supported by Grant PBZ_ KBN_122/P05/2004.

The TNBCC was supported by: a Specialized Program of Research Excellence (SPORE) in Breast Cancer (CA116201), a grant from the Breast Cancer Research Foundation, a generous gift from the David F. and Margaret T. Grohne Family Foundation. Roswell Park Cancer Institute provided samples collected and banked under the DataBank and BioRepository, supported by P30 CA 016056.

The UK Breakthrough Generations Study (UKBGS) thanks Breast Cancer Now and the Institute of Cancer Research for support and funding of the Breakthrough Generations Study, and the study participants, study staff, and the doctors, nurses and other health care providers and health information sources who have contributed to the study. We acknowledge NHS funding to the Royal Marsden/ICR NIHR Biomedical Research Centre. The UKBGS is funded by Breast Cancer Now and the Institute of Cancer Research (ICR), London. ICR acknowledges NHS funding to the NIHR Biomedical Research Centre.

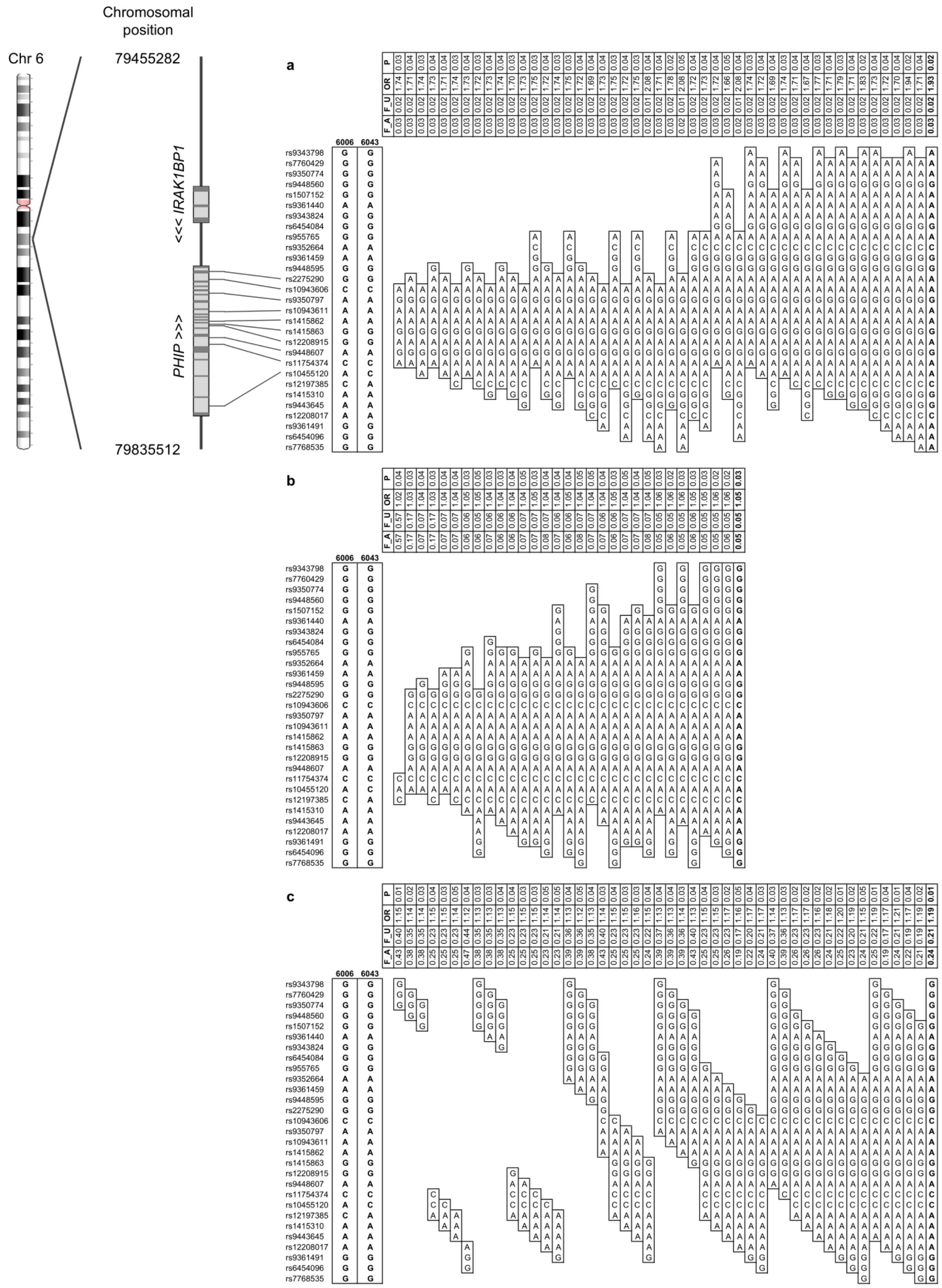

Supplementary Figure 1: Candidate risk haplotypes revealed by sliding-window analysis. Association was evaluated for haplotypes of all possible sizes (from 1 to 29) in (a) KARBAC, (b) BCAC and (c) SASBAC studies. All haplotypes with OR > 1 and p-value < 0.05 were listed with p-value, OR, estimated frequency in controls (F_U) and cases (F_A), except for the SASBAC study where only haplotypes of window size 3, 5, 10, 15, 20, 25 and 29 were shown due to space limitation. Genomic regions covered by these 29 SNPs were illustrated showing genes *IRAK1BP1* and *PHIP* whose exons and introns were indicated with dark and light grey, respectively.

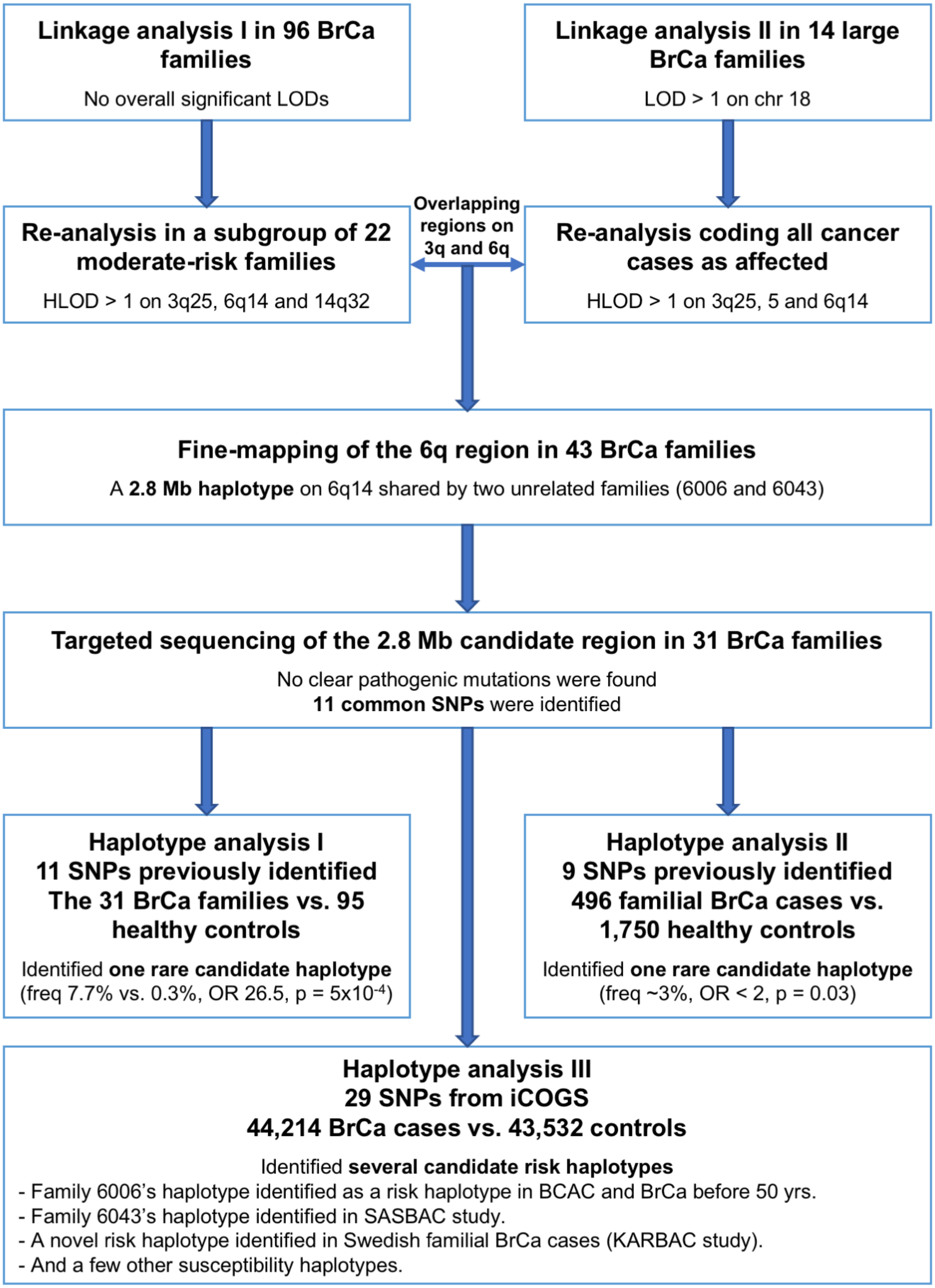

Supplementary Figure 2: Flowchart of main genetic analyses in this study.

For Supplementary Tables see in Supplementary Files.
